# Combined Optogenetic Approaches Reveal Quantitative Dynamics of Endogenous Noradrenergic Transmission in the Brain

**DOI:** 10.1016/j.isci.2020.101710

**Published:** 2020-10-21

**Authors:** Shinobu Nomura, Ludovic Tricoire, Ivan Cohen, Bernd Kuhn, Bertrand Lambolez, Régine Hepp

**Affiliations:** 1 Neuroscience Paris Seine—Institut de Biologie Paris Seine (NPS—IBPS), CNRS UMR8246, INSERM U1130, Sorbonne Université UM119, 9 quai St Bernard case 16, 75005 Paris, France; 2 Okinawa Institute of Science and Technology Graduate University, 1919-1 Tancha, Onna-son, Okinawa 904-0495, Japan

**Keywords:** Optical Imaging, Neuroscience, Cell Biology

## Abstract

Little is known about the real-time cellular dynamics triggered by endogenous catecholamine release despite their importance in brain functions. To address this issue, we expressed channelrhodopsin in locus coeruleus neurons and protein kinase-A activity biosensors in cortical pyramidal neurons and combined two-photon imaging of biosensors with photostimulation of locus coeruleus cortical axons, in acute slices and *in vivo*. Burst photostimulation of axons for 5–10 s elicited robust, minutes-lasting kinase-A activation in individual neurons, indicating that a single burst firing episode of synchronized locus coeruleus neurons has rapid and lasting effects on cortical network. Responses were mediated by β1 adrenoceptors, dampened by co-activation of α2 adrenoceptors, and dramatically increased upon inhibition of noradrenaline reuptake transporter. Dopamine receptors were not involved, showing that kinase-A activation was due to noradrenaline release. Our study shows that noradrenergic transmission can be characterized with high spatiotemporal resolution in brain slices and *in vivo* using optogenetic tools.

## Highlights

• We report all-optogenetic stimulation and imaging of NA transmission in cortex
• NA fiber stimulation elicits robust PKA activation in neurons, in slice and *in vivo*
• PKA activation involves both β1 and α2 adrenoceptors but not dopamine receptors
• Noradrenaline reuptake transporter activity greatly limits response intensity

## Graphical Abstract

**Figure:**
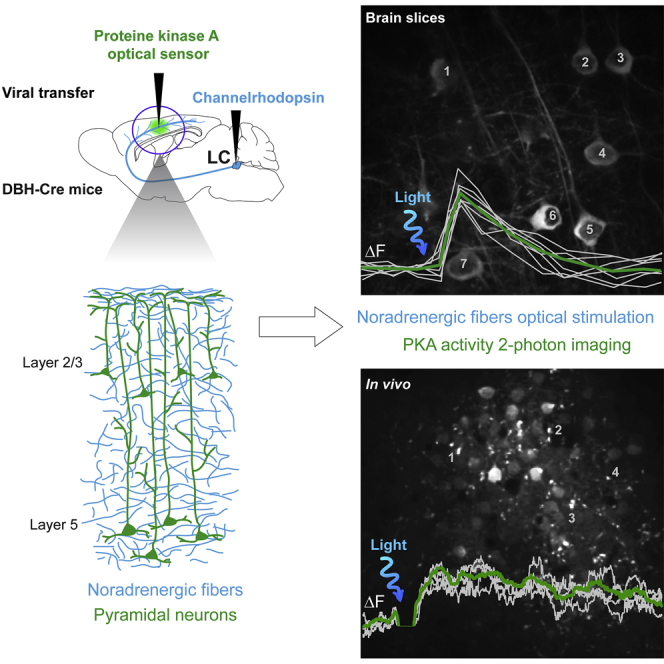

## Introduction

The catecholamines dopamine (DA) and noradrenaline (NA) are neurotransmitters that widely modulate brain circuits and behaviors. Their dysfunctions are associated with cognitive, emotional, and motor disorders, and many drugs target catecholaminergic transmission in therapy of human brain diseases. Little is known about the cellular dynamics of endogenous catecholaminergic transmission, because of the difficulty of selectively stimulating catecholaminergic fibers and because catecholamines modulate neuronal excitability but do not elicit fast and prominent membrane currents, thereby limiting the sensitivity of electrophysiological readout. However, recent reports demonstrate that optogenetic tools allow the detection of catecholamine release and of their effects at the cellular level with physiologically relevant temporal resolution in brain slices and *in vivo* (Goto et al., 2015; Ma et al., 2018; Sun et al., 2018; Feng et al., 2019; Oe et al., 2020).

NA is involved in arousal, attention, memory, and stress (Sara 2009; Benarroch 2009). It is synthetized in discrete brainstem nuclei from the amino acid tyrosine, transformed by tyrosine hydroxylase (TH) into DA, which is in turn converted into NA by dopamine beta hydroxylase (DBH). NA effects are mediated by G protein-coupled receptors, among which Gs-coupled β1-3 and Gi-coupled α2 receptors activate and inhibit adenylate cyclase, respectively, whereas Gq-coupled α1 receptors activate phospholipase C (Bylund 1992; Cotecchia 2010; Evans et al., 2010). NA-releasing fibers are widely distributed in the brain and, in the cerebral cortex, stem from neurons of the locus coeruleus (LC), whose axons innervate all layers of the entire cortical mantle (Morrison et al. 1978, 1979; Latsari et al., 2002; Nomura et al., 2014). In pyramidal cells, the major cortical neuron type, exogenous NA elicits only modest variations of membrane potential but triggers intense cAMP-protein kinase A (PKA) signals through activation of β1 adrenoceptors (McCormick et al., 1991; Castro et al., 2010; Nomura et al., 2014). Likewise, imaging of genetically encoded optical sensors for cAMP-PKA activity provides a sensitive readout of NA effects and allows detecting endogenous NA transmission events (Nomura et al., 2014; Ma et al., 2018; Oe et al., 2020).

The use of channelrhodopsin2 (ChR2) photostimulation to trigger neurotransmitter release (Nagel et al., 2003; Zhang et al., 2007) is compatible with two-photon imaging of NA effects, owing to the virtual absence of ChR2 activation upon two-photon laser scanning (Rickgauer and Tank 2009; Schoenenberger et al., 2011). We thus combined two-photon imaging of pyramidal cells expressing fluorescent PKA activity sensors with one-photon stimulation of ChR2-expressing LC fibers to characterize catecholaminergic transmission dynamics in the neocortex. We found that burst photostimulation of LC fibers resulted in transient and repeatable activation of PKA in pyramidal cells and used selective antagonists to characterize catecholamine receptors and reuptake transporters involved in this response, in brain slice and *in vivo*.

## Results

### Expression of Channelrhodopsin in LC Fibers and of AKAR3EV in Cortical Neurons

**Figure 1:**
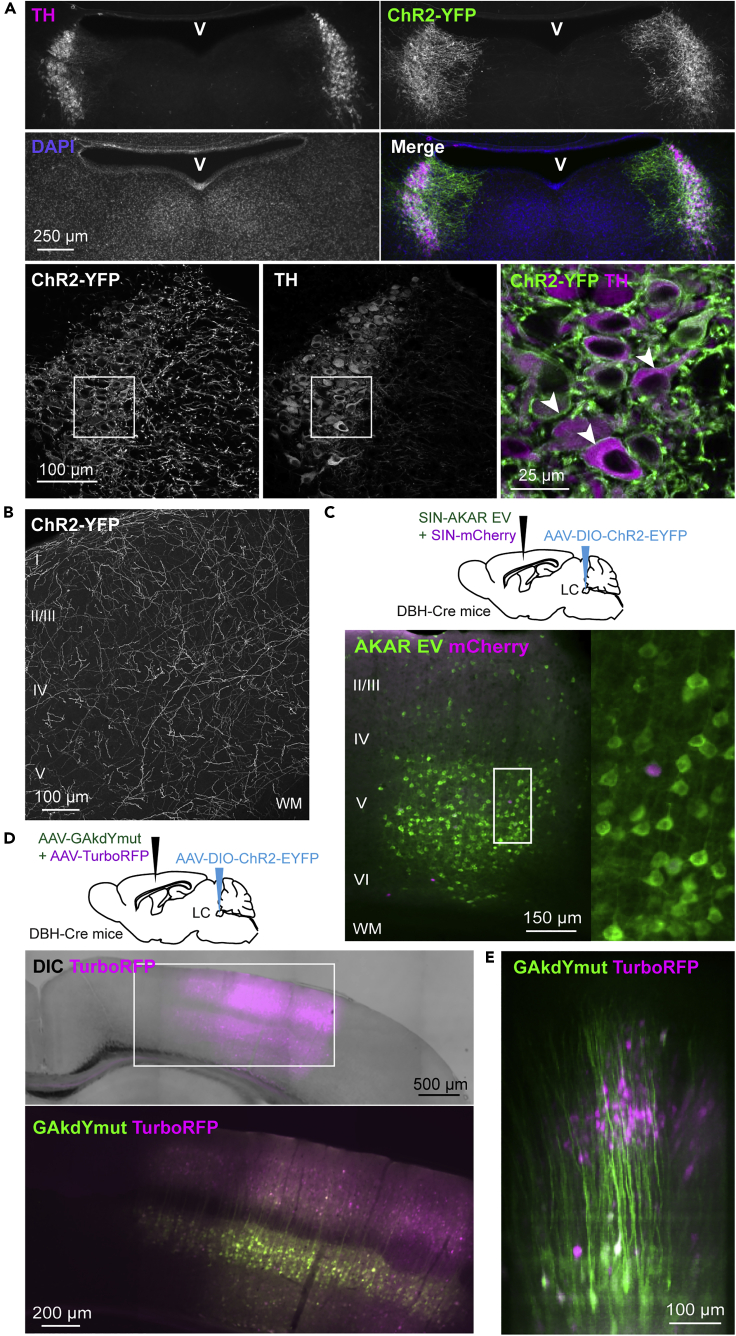
Expression of ChR2 in LC Fibers and of PKA Sensors in Cortical Neurons (A) ***Upper panels***: Immunolabelling and DAPI counterstaining of a hindbrain section shows Cre-dependent expression of ChR2-YFP in TH-positive neurons 8 weeks after bilateral AAV injection into the LC in a DBH-Cre mouse. v: lumen of the fourth cerebral ventricle. ***Lower panels***: Higher magnification shows that ChR2-YFP is selectively expressed in a large proportion of TH-positive neurons of the LC and that ChR2-YFP accumulates in plasma membranes of these neurons (as exemplified by arrowheads) (B) Immunolabelling reveals the presence of ChR2-YFP-expressing LC fibers in all layers of the parietal cortex. (C) Wide-field fluorescence of an acute slice of parietal cortex showing expression of the PKA sensor AKAR3EV in pyramidal cells 10 h after *in vivo* injection of a recombinant Sindbis virus. Co-injection of an mCherry-expressing Sindbis virus allowed visualization of the viral transduction area with minimal excitation of ChR2. (D) Images of superimposed bright-field and fluorescence (***upper***) and merged fluorescence (***lower***) of a fixed slice of parietal cortex 5 weeks after *in vivo* co-injection of AAVs expressing the GAkdYmut PKA-activity sensor and TurboRFP. (E) Merged projection of an *in vivo* two-photon 3D reconstruction of TurboRFP- and GAkdYmut-expressing neurons. Note the GAkdYmut fluorescence in extended vertical dendrites characteristic of layer V pyramidal cells.

In order to stimulate specifically LC fibers, we selectively expressed a fusion protein consisting of ChR2 and YFP in LC neurons using conditional viral transduction in DBH-Cre mice (see Methods and Nomura et al., 2014). Eight weeks after virus injection, immunohistochemistry revealed that ChR2-YFP was selectively expressed in a large proportion of DBH-positive neurons in LCs of both hemispheres (Figure 1A) but not in other catecholaminergic nuclei (not shown, but see Nomura et al., 2014). We found that 87.0 ± 2.7% of DBH-positive neurons in the LC expressed GFP (n = 214, N = 3 mice), consistent with earlier observations (Nomura et al., 2014). Conversely, only 4.9 ± 0.9% of GFP-positive cells were DBH-negative (n = 197, N = 3). ChR2-YFP was efficiently targeted to the plasma membrane of TH-positive somata (Figure 1A). In the parietal cortex, ChR2-YFP-positive LC fibers coursed throughout all layers (Figure 1B), consistent with their wide distribution in the cortical mantle (Nomura et al., 2014). These results show that the present protocol enables selective and efficient expression of ChR2-YFP in the soma and axonal processes of LC neurons.

To monitor cortical responses to photostimulation of ChR2-expressing LC fibers, we also injected recombinant viruses encoding PKA activity sensors in the parietal cortex after LC transduction (see Methods). For imaging in cortical slices, we used a Sindbis virus to express the AKAR3EV sensor (Komatsu et al., 2011). AKAR3EV is based on FRET between two GFP mutants, whose brightness facilitates the imaging in diffractive slices from myelinated adult tissue. Ten hours after virus injection, AKAR3EV fluorescence was observed throughout cortical layers II to VI, with AKAR3EV-expressing neurons observed as far as 300 μm away from the injection site (Figure 1C). For imaging cortical neurons *in vivo*, we used an AAV to express the single-chromophore, GFP-based GAkdYmut sensor (Bonnot et al., 2014), because its large dynamic range enables the detection of small changes in PKA activity and single-chromophore sensors allow two-photon imaging without the depth-dependent re-absorption problem of FRET sensors. Five weeks after AAV injection, GAkdYmut was widely expressed in layer V pyramidal neurons typically exhibiting a prominent apical dendrite (Figure 1D), allowing two-photon imaging of GAkdYmut-positive neurons in the depth of the cortex *in vivo* (Figure 1E). Co-injection of recombinant viruses encoding the red fluorophores mCherry or TurboRFP (see Methods and Figures 1C–1E) allowed visualization of viral transduction areas in cortical slices or *in vivo*, respectively, with minimal excitation of ChR2. These results show that the present approaches result in efficient cortical expression of both ChR2 in LC fibers and PKA sensors in neurons.

### Photostimulation of LC Fibers Elicits PKA Activation in Pyramidal Neurons

**Figure 2:**
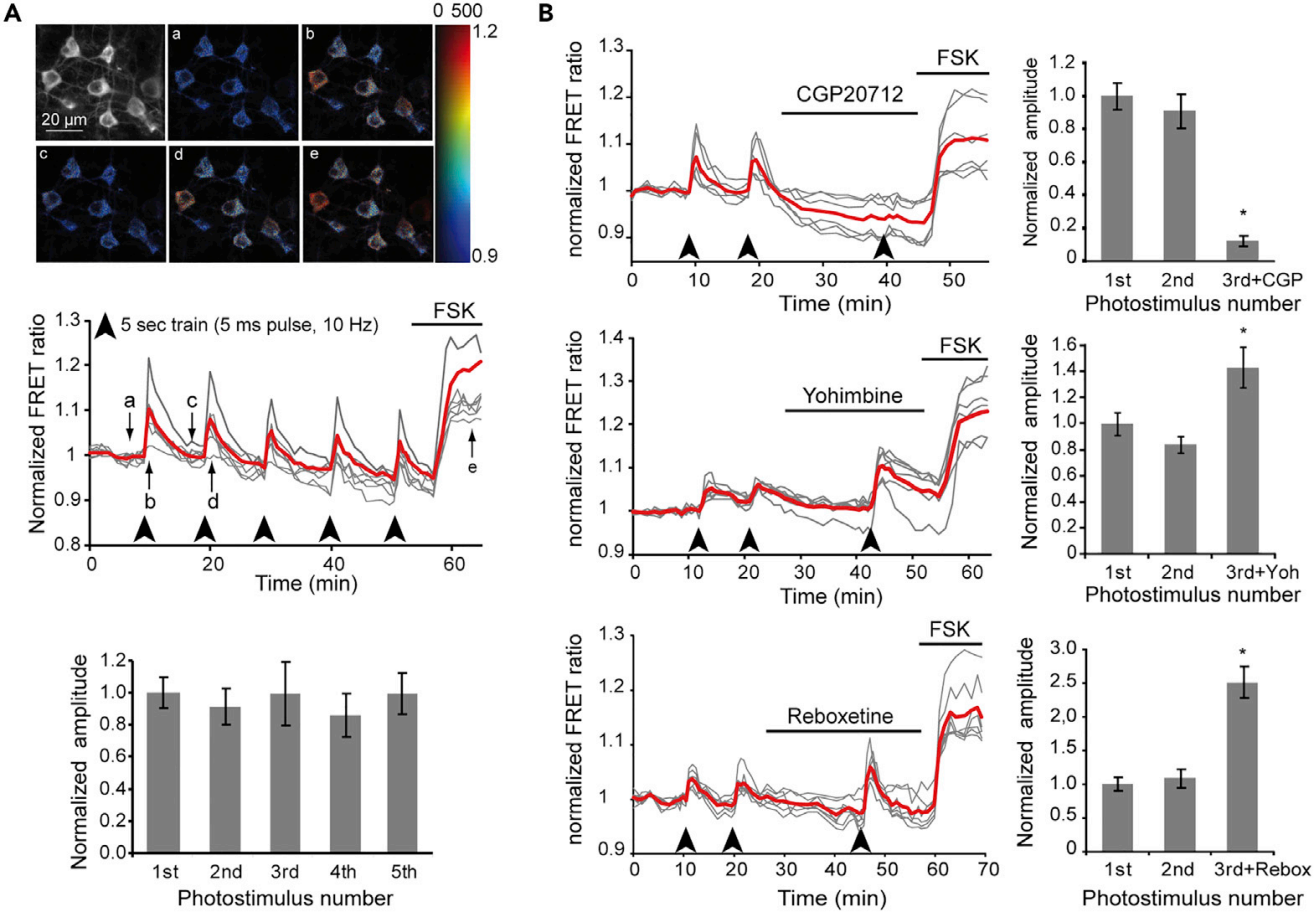
Imaging NA Transmission Triggered by Photostimulation of LC Fibers in Cortical Slices Two-photon imaging of layer V pyramidal neurons expressing the AKAR3EV sensor. (A) ***Top***: The grayscale image shows the F535 intensity and pseudocolor images show variations of the F535/F480 ratio value (coded by pixel hue) and F535 intensity (coded by pixel intensity), in pyramidal cells during the course of the experiment. ***Middle***: variations of the F535/F480 emission ratio measured at the soma of these individual pyramidal neurons (n = 7, gray traces, mean trace in red), in response to burst photostimulation (arrowheads) and to bath application of forskolin (FSK, 12.5 μM). Arrows indicate time points corresponding to pseudocolor images in the upper panel. ***Bottom***: Results obtained in n = 18 layer V pyramidal cells from N = 3 slices, from one mouse, each. Differences are not significant. Values are mean ± SEM. (B) Effects of β1 (CGP20712, 100 nM) and α2 (yohimbine, 1 μM) adrenoceptor antagonists and of the NA transporter inhibitor reboxetine (100 nM) on responses to photostimulation. ***Left***: responses of individual pyramidal neurons (gray traces, mean trace in red) in the presence or absence of drug. ***Right*,***from top to bottom*: results obtained in N = 4 and n = 18, N = 3 and n = 25, N = 5 and n = 27. ∗ Significantly different from the response to the first photostimulation. Values are mean ± SEM. ∗p < 0.05.

The Sindbis virus preferentially transduces neocortical pyramidal neurons and allows overnight expression of biosensors without conspicuous alteration of neuronal physiology (Lendvai et al., 2000; Gervasi et al., 2007; Drobac et al., 2010; Hu et al., 2011). We imaged PKA dynamics in AKAR3EV-expressing layers II/III and V pyramidal cells from cortical slices, which both respond to exogenous catecholamines (Nomura et al., 2014), and focused primarily on layer V pyramidal neurons. In a series of pilot experiments, photostimulation patterns were tested for their ability to evoke PKA signals, based on the *in vivo* burst firing of LC neurons that discharge action potentials at a maximal frequency of 10 Hz (Aston-Jones and Bloom, 1981a; 1981b), which is compatible with ChR2 kinetic properties (Mattis et al., 2011). We thus applied trains of light pulses of variable length, at a maximal frequency of 10 Hz, for different overall durations. Most protocols elicited transient PKA activation (see examples in Figure S1). However, photostimulation consisting of 5-ms light pulses applied at 10 Hz during 5 s yielded robust and reproducible responses (Figure 2A) and was used in all subsequent experiments in brain slices. Unless otherwise stated, responses were measured at the soma of pyramidal neurons. At the end of photostimulation series, saturation of the AKAR3EV sensor was obtained by bath application of a maximally effective concentration of the adenylate cyclase activator forskolin (FSK, 12.5 μM; Gervasi et al., 2007) (Figure 2A). Photostimulation induced clearly detectable peaks of PKA activity in 93 ± 7% of individual layer II/III (n = 34 of 37, N = 4 slices from four mice) and in 86 ± 14% of individual layer V (n = 18 out of 21, N = 3 slices from three mice) pyramidal neurons. Responses were transient and were consistently observed at each stimulus upon repetitive stimulation (interval between photostimulation trains, ~10 min, see examples in Figures 2A and S2). Despite individual variability (Figure S3), mean responses of layer V pyramidal cells to five successive photostimulus trains exhibited roughly stable amplitudes that ranged between 86 ± 14% and 100 ± 10% of the first response (n = 18 cells, N = 3 slices from three mice; Figures 2A and S3 and Table 1). Essentially similar results were obtained in the dendrites of these neurons (Figure S4), although larger mean amplitudes were observed in dendrites than in somata (Figure S4 and Table 1). The increase of the FRET ratio elicited by the first photostimulation in the soma of these neurons was 7.4 ± 1.2%, which represents roughly one-third of their response to FSK (25.7 ± 2.0% increase of FRET ratio, Table 1). For comparison, PKA responses of cortical pyramidal cells to bath application of a saturating dose of NA reach 60% of the response to FSK (Nomura et al., 2014), corresponding to only twice the response amplitude measured here upon LC fiber photostimulation. This suggests that a significant proportion of the catecholamine receptors expressed in pyramidal cells are activated upon burst photostimulation of LC fibers. Responses of layer V pyramidal cells peaked within 1.5 min after photostimulation and decayed with time constants ranging from 140 ± 15 to 178 ± 18 s (Table 1). Response kinetics did not significantly differ between the five photostimulation trains (Table 1). Essentially similar results were obtained in layer II/III pyramidal neurons, except that response amplitude gradually declined upon repetitive stimulation (Figure S2 and Table 1). We thus focused on somata of layer V pyramidal neurons in subsequent brain slice experiments. These results suggest that selective photostimulation of cortical LC fibers triggers the release of endogenous catecholamines, which robustly activate cAMP/PKA signaling in pyramidal neurons, thereby allowing the pharmacological characterization of their responses.

**Table 1:** Properties of PKA Responses Obtained in Pyramidal Neurons from Cortical Slices upon Five Consecutive Photostimulation Episodes

Photostimulation	1		2		3		4		5		Forskolin	
Cortical Layer	II/III	V	II/III	V	II/III	V	II/III	V	II/III	V	II/III	V
Amplitude in somata (ΔR/R0%)	7.3 ± 0.5 (n = 34)	7.4 ± 1.2 (n = 18)	6.0∗ ±0.4 (n = 34)	6.1 ± 0.9 (n = 18)	4.9∗† ±0.3 (n = 34)	5.6 ± 0.8 (n = 18)	4.6∗† ±0.4 (n = 34)	5.7 ± 0.9 (n = 18)	4.5∗† ±0.3 (n = 34)	6.4 ± 1.0 (n = 18)	20.5 ± 0.9 (n = 34)	25.7 ± 2.0 (n = 18)
Normalized amplitude in somata (% first stim)	100 ± 7.3 (n = 34)	100 ± 9.8 (n = 18)	77.6∗ ±5.6 (n = 34)	91.4 ± 11.5 (n = 18)	58.36∗ ±5.6 (n = 34)	99.4 ± 19.9 (n = 18)	59.7∗ ±5.0 (n = 34)	85.8 ± 13.6 (n = 18)	53.1∗ ±4.7 (n = 34)	99.6 ± 12.9 (n = 34)	NM	NM
Amplitude in dendrites (ΔR/R0%)	NM	10.2 ± 1.8 (n = 31)	NM	8.5 ± 1.5 (n = 31)	NM	8.1 ± 1.5 (n = 31)	NM	8.2 ± 1.5 (n = 31)	NM	7.9 ± 1.4 (n = 31)	NM	28 ± 5 (n = 31)
Time to peak range (s)	[60–90] (n = 24)	[60–90] (n = 17)	[60–90] (n = 24)	[60–90] (n = 17)	[60–90] (n = 24)	[60–90] (n = 17)	[60–90] (n = 24)	[60–90] (n = 16)	[60–90] (n = 24)	[60–90] (n = 17)	NM	NM
Decay (τ in s)	221.7 ± 17.1 (n = 20)	178.4 ± 17.7 (n = 12)	220.8 ± 26.4 (n = 20)	147.4 ± 10.6 (n = 12)	223.6 ± 27.4 (n = 19)	172.2 ± 27.8 (n = 12)	232.9 ± 24.4 (n = 20)	159.7 ± 15.6 (n = 12)	212.6 ± 28.3 (n = 20)	140.5 ± 15.2 (n = 12)	NM	NM

Each photostimulation episode consisted of 5-ms light pulses at 10 Hz for 5 s AKAR3EV imaging: R = F535/F480. Time to peak refers to the time between the beginning of the photostimulation and the maximum of the response. The value has been calculated for each response and the mean value has been replaced by a 30-s-long time interval to take in account the acquisition rate (see Methods). The decay time τ was calculated by fitting the decay of the response with a single exponential. Time to peak and decay were calculated for responses in somata. Experiments were performed on four and three independent brain slices for layers II/III and V, respectively. Values are mean ± SEM. ∗ and † indicate values significantly different (p < 0.05) from those obtained for the first and second stimulation, respectively. NM, not measured.

### PKA Dynamics Evoked by LC Fibers Photostimulation Are Mediated by NA Receptors

LC fibers can release both NA and DA in the forebrain (Devoto et al. 2001, 2005, 2008, 2015; Kempadoo et al., 2016), and both NA and DA receptors are widely expressed in the neocortex, beyond the restricted distribution of dopaminergic fibers (Ariano and Sibley 1994; Khan et al., 1998; Luedtke et al., 1999; Rivera et al., 2008; Oda et al., 2010; Nomura et al., 2014). In the parietal cortex, DA fibers are scarce and restricted to layer VI, but Gs-coupled D1-5 and Gi-coupled D2-like DA receptors are nonetheless functionally expressed and coupled to cAMP/PKA signaling in layers II-V pyramidal cells (Nomura et al., 2014). We thus characterized receptors involved in light-induced responses using specific antagonists of Gs- and Gi-coupled NA and DA receptors. Following two photostimulation trials in control condition to assess the stability of the PKA response, the third photostimulation was performed in the presence of antagonist pre-applied for at least 10 min. Response amplitudes thereafter are expressed as percentage of the first response amplitude. Perfusion of the specific β1-adrenergic receptor antagonist CGP20712 (100 nM) almost completely prevented PKA activation (Figure 2B and Table 2). The amplitude of the residual response was 12 ± 3% of the first response (N = 4, n = 18). Conversely, application of the D1/D5 receptor antagonist SCH23390 (1 μM) did not significantly modify the response (117 ± 10%, N = 3, n = 25; Table 2). These results indicate that light-induced PKA activation in pyramidal cells was essentially mediated by β1-adrenergic receptors, without detectable contribution of the D1/D5 receptors functionally expressed in these cells (Nomura et al., 2014). We next tested whether responses to photostimulation also involve Gi-coupled receptors by applying the α2 adrenoceptor antagonist yohimbine (1 μM) and the D2-like dopaminergic receptor antagonist haloperidol (10 μM). Application of yohimbine resulted in an increase of response amplitude (143 ± 15%, N = 3, n = 21; Figure 2B and Table 2), whereas haloperidol did not significantly alter the response (87 ± 10%, N = 3, n = 21; Table 2). Hence, PKA activation upon photostimulation of LC fibers is mediated by Gs-coupled β1 adrenoceptors and is dampened by co-activation of Gi-coupled α2 adrenoceptors. This suggests that NA released by LC fibers activates both receptor types in pyramidal cells, as also observed upon application of exogenous NA (Nomura et al., 2014). Conversely, these results rule out the involvement of DA receptors in PKA responses of pyramidal cells to burst photostimulation of LC fibers.

**Table 2:** Pharmacological Properties of Responses to Photostimulation of LC Fibers in Cortical Slices

Photostimulation	1 (Control)	2 (Control)	3 (+Drug)	
Amplitude (% 1st stimulation)	100 ± 8% (n = 18)	91 ± 10% (n = 18)	12.4 ± 3.2% (n = 18)	CGP20712 (100 nM) β1 antagonist
Amplitude (% 1st stimulation)	100 ± 9% (n = 21)	84 ± 6% (n = 21)	**143∗† ± 15% (n = 21)**	Yohimbine (1 μM) α2 antagonist
Time to peak (s)	[60–90] (n = 19)	[60–90] (n = 19)	[60–90] (n = 19)	
τ decay (s)	264.1 ± 32.4 (n = 13)	256.6 ± 43.0 (n = 13)	283.4 ± 36.9 (n = 13)	
Amplitude (% 1st stimulation)	100 ± 5.8% (n = 25)	88.2 ± 4.1% (n = 25)	117 ± 10.8% (n = 25)	SCH23390 (1μM) D1/D5 antagonist
Amplitude (% 1st stimulation)	100 ± 12.2% (n = 21)	88 ± 10.2% (n = 21)	86.8 ± 10.5% (n = 21)	Haloperidol (10 μM) D2-like antagonist
Amplitude (% 1st stimulation)	100 ± 10% (n = 27)	109 ± 12% (n = 27)	**251∗† ± 23% (n = 27)**	Reboxetine (100 nM) NET inhibitor
Time to peak (s)	[60–90] (n = 23)	[60–90] (n = 27)	**[90–120]∗† (n = 27)**	
τ decay (s)	184.9 ± 31.4 (n = 12)	195.3 ± 23.0 (n = 11)	189.2 ± 29.6 (n = 18)	
Amplitude (% 1st stimulation)	100 ± 8.2% (n = 20)	87.7 ± 8.5% (n = 20)	85.7 ± 8.2% (n = 20)	GBR12783 (100 nM) DAT inhibitor
Amplitude (% 1st stimulation)	100 ± 10% (n = 24)	85.6 ± 8.5% (n = 24)	89.7 ± 8.4% (n = 24)	corticosterone (100 μM) OCT inhibitor

Photostimulation consisted of 5-ms light pulses at 10 Hz for 5 s, and PKA activity was measured in somata of layer V pyramidal cells from three to five independent brain slices. Values are mean ± SEM. ∗ and † correspond to values significantly different (p < 0.05) from the values obtained for the first and second stimulation, respectively.

### Cortical NA Transmission Is Critically Controlled by Catecholamine Reuptake

The clearance of released catecholamines involves their recapture into catecholaminergic axons or neighboring cells by various transporters showing low selectivity for NA versus DA (Grundemann et al., 1998; Torres et al., 2003). We tested the role of the NA transporter (NET), DA transporter (DAT), and extraneuronal transporters of the organic cation transporter (OCT) family in light-induced PKA responses. We found that application of the specific NET inhibitor reboxetine (100 nM) largely increased the response to photostimulation, whose amplitude was more than twice that of the response to the first photostimulation (251 ± 23%, N = 5, n = 27; Figure 2B). This was accompanied by an increase of the response time to peak, without significant modification of decay kinetics (Table 2). In contrast, neither the specific DAT inhibitor GBR12783 (100 nM) nor the OCT inhibitor corticosterone (100 μM, Grundemann et al., 1998) significantly modified the amplitude of the PKA response (GBR12783: 86 ± 8%, N = 3, n = 20; corticosterone: 90 ± 8%, N = 3, n = 24; Table 2). These results indicate that the effects of NA released upon burst photostimulation of LC fibers are strongly reduced by reuptake through NET transporters. Conversely, DAT and OCT were not involved in shaping cortical catecholaminergic transmission from LC fibers.

### Cortical NA Transmission *In Vivo*

**Figure 3:**
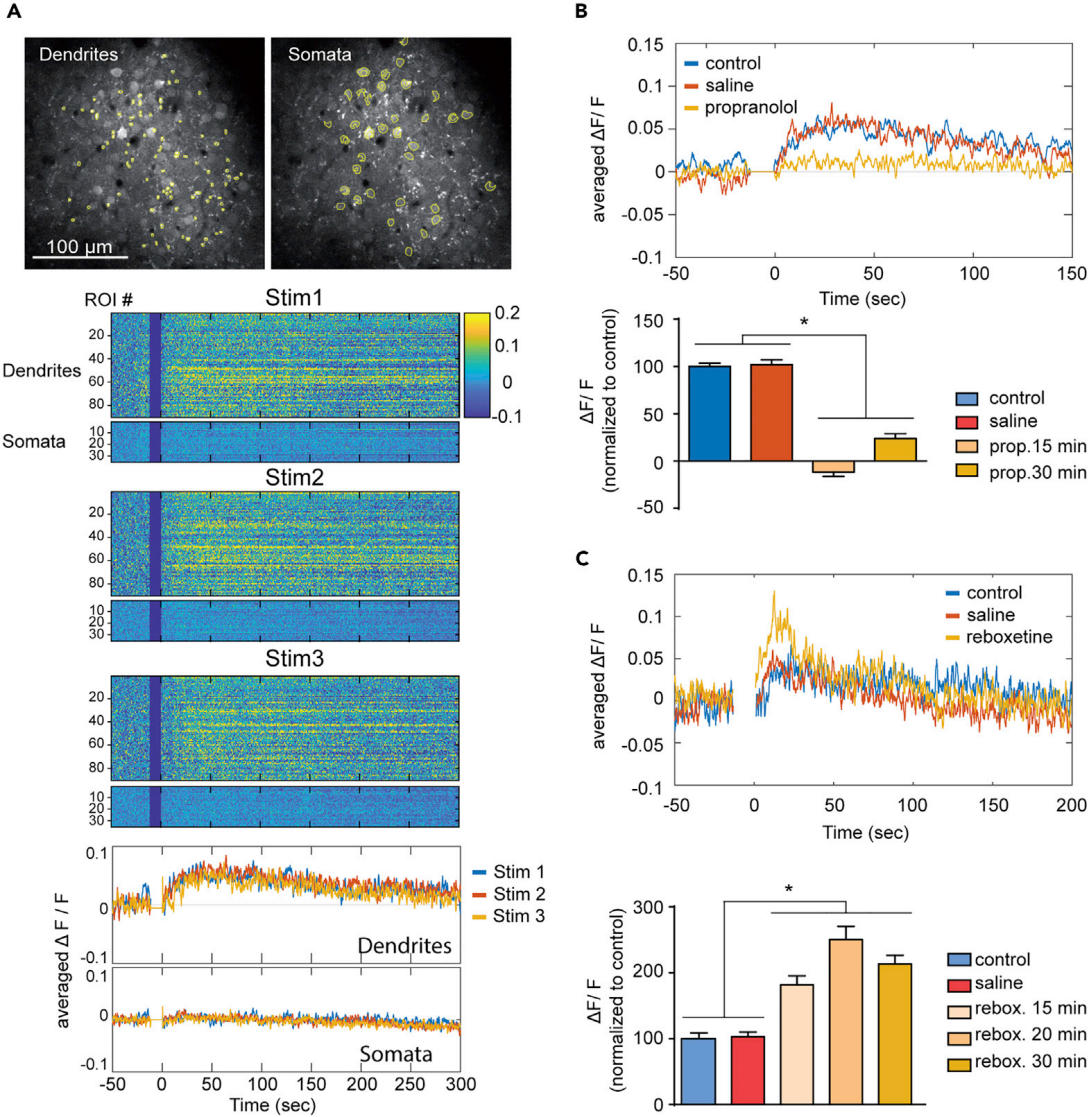
Imaging Cortical NA Transmission *In Vivo* upon LC Fibers Photostimulation Two-photon imaging of cortical neurons expressing the GAkdYmut sensor. (A)***Top*:** The grayscale images (275 × 275 μm) show the two-photon imaging plane (168 μm below dura in layer 2/3) with yellow circles delineating ROIs around GAkdYmut-positive dendrites and somata. ***Middle*:** Raster plots show GAkdYmut fluorescence intensity (calibration bar: ΔF/F) of these ROIs before and after each of three consecutive bursts of photostimulation (darker vertical bar, 5 ms pulses at 20 Hz for 10 s).***Bottom*:** Traces show averaged fluorescence intensity of the same ROIs. (B)***Top***: Example of the averaged response to photostimulation (100 dendritic ROIs), which persisted following i.p. injection of saline but was abolished 15 min after i.p. injection of the β-adrenoceptor antagonist propranolol (4 mg/kg).***Bottom***: Results obtained in three mice for 330 dendritic ROIs. Values are mean ± SEM. ∗p < 0.05. (C) Traces exemplify the increase of the response to photostimulation in 45 dendritic ROIs 15 min after reboxetine (10 mg/kg) i.p. injection. The bar graph shows the results obtained in three mice for 190 dendritic ROIs. Values are mean ± SEM. ∗p < 0.05.

In order to validate, in the intact neocortex, the main results obtained in brain slice, we used *in vivo* photostimulation of LC fibers and imaging of pyramidal neurons expressing the GAkdYmut PKA sensor and TurboRFP (see Methods and Figure 1). We monitored GAkdYmut fluorescence intensity in dendrites and somata during time windows corresponding to three consecutive bursts of photostimulation (interval between bursts, 5 min, Figure 3A). The photostimulation pattern used in slices (5 ms pulse at 10 Hz for 5 s) failed to evoke consistent responses *in vivo* (Figure S5), presumably because side illumination through the cranial window (see Methods) and tissue light scattering properties resulted in suboptimal ChR2 activation in depth of the cortex. We thus increased photostimulation (5 ms at 20 Hz for 10 s) and observed clear responses, which consisted in transient GAkdYmut fluorescence increases in dendrites (see example in Figure 3A). Mean responses of dendritic ROIs reached 5.7 ± 0.7% of baseline GAkdYmut fluorescence (n = 960 ROIs, 12 stimulation trials performed in N = 6 mice). No change in dendritic TurboRFP fluorescence was observed in the same experiments (Figure S6). In order to establish a criterion to select regions of interest (ROIs) for further quantitative analyses of responses, fluorescence changes of each dendritic ROI were submitted to a statistical test to determine their individual responsiveness to photostimulation (see Methods). Only ROIs whose post-stimulus fluorescence significantly differed from baseline were considered as responsive. Among the 90 dendritic ROIs shown in Figure 3A, we found that 60, 67, and 58 ROIs significantly responded to the first, second, and third successive stimulation trials, respectively, with 42.2% of these 90 ROIs responding to all three photostimulation trials (Figure S7). Responses of individual dendritic ROIs showed ROI-to-ROI and stimulus-to-stimulus variability (Figure S7), but averaged responses of pooled dendritic ROIs had stable amplitude upon repetitive photostimulation (see example in Figure 3A). Although responses to photostimulation were also detectable in somatic ROIs (Figure 3A), they were much less frequent compared with dendrites. Indeed, using the same statistical criterion as above, we found that 10, 7, and 8 of 35 somatic ROIs significantly responded to the first, second, and third successive stimulation trials, respectively, with only 5.7% of these 35 somatic ROIs responding to all three photostimulation trials (Figure S8). Furthermore, somatic fluorescence changes were of much smaller amplitude than dendritic ones (p < 0.001, Mann Whitney test, Figure S8), consistent with the differential responsiveness of these subcellular domains to β-adrenoceptor-induced cAMP/PKA signaling (Castro et al., 2010). When considering all responsive dendritic ROIs over the three trials shown in Figures 3A and S7, fluorescence increases were larger than 2% of baseline in 99.5% of these ROIs (n = 59 + 67+58 = 184 of 185 traces, see above). Conversely, among fluorescence responses higher than 2% (225 traces), 81.8% of traces passed our statistical test. Thus, in the rest of this *in vivo* study, we only considered dendritic fluorescence changes larger than 2% of baseline for analyses of light-induced responses. Among ROIs imaged in control conditions (n = 960 ROIs, 12 stimulation trials performed in N = 6 mice), those showing fluorescence changes >2% (76 ± 3% of ROIs) were selected to calculate mean response kinetics, yielding onset (τ = 14.7 ± 4.5 s) and decay (τ = 154.1 ± 31.0 s) time constants. We then imaged GAkdYmut fluorescence in dendrites to characterize the pharmacological properties of responses to effective photostimulation (5 ms pulse, 20 Hz, 10 s) of LC fibers *in vivo*. In each experiment, photostimulation was performed first in control condition, then 15 min after i.p. injection of saline, and finally after i.p. injection of drug. Response amplitudes remained stable after saline injection (103 ± 4% of control, n = 520 ROIs, N = 6) but markedly changed after drug administration (Figures 3B and 3C). Indeed, the response was virtually abolished 15 min after injection of the β-adrenoceptor antagonist propranolol (4 mg/kg, - 11 ± 5% of control, n = 330 ROIs, N = 3) and only partially recovered 30 min after propranolol injection (24 ± 5% of control, n = 330 ROIs, N = 3, Figure 3B). Conversely, response amplitude was largely enhanced 15 min after injection of reboxetine (10 mg/kg, 182 ± 14% of control, n = 190, N = 3), reaching a maximum 20 min after injection (250 ± 20% of control, n = 125, N = 2) before declining 30 min after reboxetine treatment (214 ± 13% of control, n = 190, N = 3, Figure 3C). These results confirm, in the cortex *in vivo*, that burst photostimulation of LC fibers triggers the release of NA, which activates PKA signaling in pyramidal neurons via β-adrenoceptors, and whose effects are limited by reuptake through NET transporters.

## Discussion

We combined imaging of PKA sensors expressed in pyramidal neurons with photostimulation of ChR2-expressing LC fibers in the neocortex, in acute slices and *in vivo*. We found that burst photostimulation of LC fibers elicited fluorescence changes of the sensors, indicative of PKA activation in somata and dendrites of individual pyramidal cells. These responses were transient, and their amplitude was stable upon repetitive stimulation. The use of selective antagonists showed that responses were mediated by β1 adrenoceptors and dampened by co-activation of α2 adrenoceptors, but did not involve DA receptors, showing that NA was the major catecholamine released from LC fibers. Responses to photostimulation were largely increased upon NET inhibition, indicating that the clearance of NA released from LC fibers is primarily achieved by reuptake through this transporter. Our results demonstrate that the present optogenetic tools provide sensitive and reliable means to analyze quantitatively the dynamics of NA transmission in the brain at cellular and subcellular levels.

### Burst Photostimulation of LC Fibers Triggers PKA Activation

Biosensors of the AKAR series, which include AKAR3EV and GAkdYmut, are based on conformational changes upon phosphorylation of a PKA substrate domain and selectively report PKA activity *in cellulo* (Zhang et al. 2001, 2005; Allen and Zhang 2006; Gervasi et al., 2007; Hu et al., 2011; Bonnot et al., 2014). Our results thus indicate that burst photostimulation of LC fibers evoked robust cAMP/PKA signaling transients, clearly detectable in real time at the level of single pyramidal neurons and their dendrites, in slices and *in vivo*. Light-induced responses in slices reached one-third the amplitude of responses to FSK, indicating that NA released by LC fibers is able to activate a significant proportion of somatodendritic β1 adrenoceptors present at the membrane of pyramidal neurons, despite reuptake by NET. For comparison, the maximal effect of exogenous NA is about two-thirds of the FSK effect on cortical pyramidal cells (Nomura et al., 2014), suggesting that NA released upon burst photostimulation in slices reached the membrane of these neurons at a mean concentration close to its EC50 (25 nM, Nomura et al., 2014). Smaller light-induced responses were observed *in vivo*, reaching only ~8% of the FSK effect previously measured in slice (74% increase of GAkdYmut fluorescence, Bonnot et al., 2014), consistent with less efficient photostimulation of LC fibers *in vivo*. Indeed, in these latter conditions (see Methods), illumination angle, light reflection/refraction at cranial glass window interfaces, as well as light scattering and absorption through blood-perfused tissue likely resulted in low illumination intensity of LC fibers in the imaging field in layer II/III. Dendritic responses were larger than somatic responses both in slices and *in vivo*. The differential responsiveness of these cellular domains to β-adrenoceptor-induced cAMP/PKA signaling in cortical pyramidal cells is documented and attributed to the effect of phosphodiesterase type 4 that prevents diffusion of cAMP from the submembrane space to the cytosol, thereby favoring domains with a high surface to volume ratio, such as dendrites (Castro et al., 2010). Responses were time-locked on the photostimulus, and imaging at high temporal resolution *in vivo* (6 Hz) revealed rapid onset (τ = 15 s) and slower decay (τ = 154 s) kinetics, in good correspondence with our results in brain slices. Prior observations indicate that the time course of light-induced responses of the biosensors followed that of the PKA signal in pyramidal cells: (1) responses of AKAR probes to cAMP photo-uncaging peak within 30 s (Zhang et al., 2005; Gervasi et al., 2007), (2) responses of AKAR probes and of cAMP biosensors exhibit similar onset kinetics upon activation of Gs-coupled receptors (Castro et al., 2010), (3) relaxation kinetics of AKAR probes (i.e., their dephosphorylation by cellular phosphatases) are faster than the decay of the present responses to photostimulation (Allen and Zhang 2006; Dunn et al., 2006; Gervasi et al., 2007). Hence, our results indicate that burst activation of LC fibers for a few seconds rapidly triggers robust, minutes-lasting cAMP/PKA signals in cortical pyramidal cells.

### PKA Dynamics Evoked by Burst Photostimulation Rely on NA but Not DA Transmission

The activation of PKA in pyramidal cells was essentially mediated by β1 adrenoceptors, consistent with NA release from LC fibers and with the effect of exogenous NA (Nomura et al., 2014). The α2 antagonist yohimbine increased the amplitude of light-induced responses by 140%, an effect larger than that observed on responses to exogenous NA (117%, Nomura et al., 2014). This suggests that recruitment of α2 adrenoceptors dampens the response both by inhibiting adenylate cyclase in pyramidal cells and by acting at LC fibers to decrease NA release, as classically described (Starke 2001). The NET inhibitor reboxetine dramatically enhanced the amplitude of light-induced responses but did not change their decay kinetics. This indicates that NA reuptake by NET limits the number of receptors activated upon NA release by minimizing the spatial extent of NA diffusion but is not involved in limiting the duration of NA transmission events. We exclude a role of DAT and OCT in shaping light-induced responses but cannot rule out a contribution of the corticosterone-insensitive uptake2 transporter PMAT (Engel et al., 2004; Engel and Wang 2005) in NA clearance, since the fluorescence of its antagonist decynium-22 (Inyushin et al., 2010) precluded its use in our imaging study.

Devoto et al. (2001, 2005, 2008, 2015) have shown that electrical stimulation of the LC *in vivo* results in cortical release of both NA and its biosynthetic precursor DA. However, although both D1/D5 and D2-like receptors are functionally coupled to adenylate cyclase in pyramidal cells (Nomura et al., 2014), we failed to detect their involvement in PKA responses to photostimulation in cortical slices. This discrepancy presumably relates to the low potency of DA (notably a ~20-fold higher EC50 than NA) at stimulating PKA in cortical pyramidal cells (Nomura et al., 2014), which suggests in turn two hypotheses regarding DA transmission from cortical LC fibers. (1) DA transmission may occur through DA receptors preferentially coupled to phospholipase C, as documented for D1, D2, and D5 receptors (reviewed by Beaulieu and Gainetdinov 2011), thereby escaping our present detection using PKA biosensors. (2) Alternatively, burst photostimulation in cortical slices may elicit only minimal DA release, whereas intense and prolonged activation of LC fibers *in vivo* (as performed by Devoto et al.) may favor DA release and thus lead to the recruitment of DA receptors coupled to adenylate cyclase. Indeed, in these latter conditions, DA may accumulate in vesicles pending depletion of vesicular DBH that is co-released with NA (Weinshilboum et al., 1971) or may be released by reverse transport from the axoplasm upon inversion of ion gradients (Levi and Raiteri 1993; Sitte and Freissmuth 2010). It is noteworthy that our *in vivo* data, i.e., the complete block of light-induced responses by the β-adrenergic antagonist propranolol, do not rule out DA receptor activation, since propranolol also prevents D1/D5 receptor-mediated effects of exogenous DA on pyramidal cells (Nomura et al., 2014). Hence, further work is required to assess and characterize NA and DA co-transmission from cortical LC fibers.

### Phasic Noradrenergic Transmission and Burst Firing of LC Neurons

The firing of LC neurons consists either of single spikes emitted at low frequency (tonic mode) or of bursts of action potentials (phasic mode) that involve synchronization of the population of LC neurons (Aston-Jones and Bloom, 1981a; 1981b). The burst firing of LC neurons is associated with a peak of NA release and has important behavioral correlates (Florin-Lechner et al., 1996; Aston-Jones and Cohen 2005). Our results obtained upon bulk photostimulation indicate that a single episode of synchronized burst firing in LC neurons has rapid and long-lasting effects on the cortical network, and that phasic NA transmission operates with higher intensity and temporal precision than generally thought of a neuromodulatory system relying on volume transmission. Light-induced responses exhibited cell-to-cell and dendrite-to-dendrite variability, as observed upon exogenous NA application (Nomura et al., 2014), presumably reflecting variations in distance to NA release sites, involvement of α2 adrenoceptors, and basal level of cAMP/PKA activity. Nonetheless, population responses were robust and reproducible. Gs-coupled receptors enhance neuronal excitability by decreasing PKA-sensitive membrane potassium currents with onset kinetics similar to those of AKAR probes responses (Gervasi et al., 2007; Hu et al., 2011). Based on these latter studies, the present light-induced PKA responses (~30% of FSK effect) would translate into a ~50% reduction of PKA-sensitive potassium currents in pyramidal neurons. Hence, a single episode of synchronized burst firing in LC neurons likely induces a rapid and substantial increase of the excitability of pyramidal neuron populations with potentially important acute and long-term effects on the function of the cortical network.

### Imaging the Cellular Dynamics of Catecholaminergic Transmission

The detectability of endogenous catecholaminergic transmission *in vivo* has been recently demonstrated using fluorescent cAMP or PKA activity sensors expressed in neurons or glia (Goto et al., 2015; Ma et al., 2018; Oe et al., 2020). Here we show that two-photon imaging of FRET or single-wavelength PKA sensors allows the quantitative analysis of key properties (intensity, kinetics, receptors, and reuptake transporters) of NA transmission events in cortical neurons and their dendrites, in brain slice and *in vivo*. Our results confirm that any optical sensor of cAMP/PKA signaling is suitable for the imaging of endogenous NA transmission, pending sufficient dynamic range. In this regard, the bright AKAR3EV FRET sensor proved useful for the imaging in diffractive adult slice, despite smaller dynamic range than GAkdYmut (ΔR/R0: ~25% versus ΔF/F0: 74%, respectively, in response to FSK in similar experimental conditions; see Table 1 and Bonnot et al., 2014). Conversely, GAkdYmut enabled fast acquisition (375 × 375 μm frame at 6.2 Hz) to analyze PKA response kinetics with high resolution *in vivo*. This single-wavelength green sensor is well adapted to two-photon imaging, as fluorescence can be collected in its entire emission spectrum for increased sensitivity, and is easily compatible with other sensors for multicolor imaging, as exemplified here by the dual monitoring of GAkdYmut and TurboRFP *in vivo*. It is thus likely that GAkdYmut can be combined with single-wavelength cAMP or Ca^2+^ red sensors with large dynamic range (e.g., Dana et al., 2016; Ohta et al., 2018; Oe et al., 2020) for future examination of endogenous catecholaminergic transmission dynamics and their impact on network electrical activity.

### Limitations of the Study

The amplitude and kinetics of cortical NA transmission events occurring upon spontaneous burst firing of LC neurons in awake mice may largely differ from those presently reported using optogenetic stimulation in conditions of low network activity found in slices and anesthetized animals. Another limitation concerns the use of biosensors for PKA activity, whose readout of receptor binding/unbinding is presumably delayed as compared with sensors for upstream signaling events (e.g., cAMP changes) and which cannot detect the possible recruitment of Gq-coupled α1 adrenoceptors (Cotecchia 2010) and DA receptors (Beaulieu and Gainetdinov 2011) elicited by LC fibers stimulation.

### Resource Availability

#### Lead Contact

Further Information and requests for resources and reagents should be directed to and will be fulfilled by the Lead Contact, Bertrand Lambolez (bertrand.lambolez@upmc.fr).

#### Material Availability

This study did not generate new unique reagents.

#### Data and Code Availability

The data and codes reported in this study are available from the Lead Contact on request.

## Methods

All methods can be found in the accompanying Transparent Methods supplemental file.

## Supplemental Information

Document S1. Transparent Methods and Figures S1–S8
