# Supplementary material for: Combined Optogenetic Approaches Reveal Quantitative Dynamics of Endogenous Noradrenergic Transmission in the Brain
Source: iScience. 2020 Oct 21;23(11):101710. doi: 10.1016/j.isci.2020.101710 (PMC7645030; doi:10.1016/j.isci.2020.101710)
Supplement: Document S1. Transparent Methods and Figures S1–S8 [file mmc1.pdf]

**iScience, Volume *23***

## Supplemental Information

# Combined Optogenetic Approaches Reveal Quantitative Dynamics of Endogenous Noradrenergic Transmission in the Brain

**Shinobu Nomura, Ludovic Tricoire, Ivan Cohen, Bernd Kuhn, Bertrand Lambolez, and Régine Hepp**

**The supplement contains:**

- **Supplementary Figure 1.** Test of different patterns of LC fibers photostimulation in cortical slices.
- **Supplementary Figure 2.** Responses of layer II/III pyramidal neurons to photostimulation of LC fibers in cortical slices.
- **Supplementary Figure 3.** Variability of layer V pyramidal neurons responses to photostimulation of LC fibers in cortical slices.
- **Supplementary Figure 4.** Comparison between dendritic and somatic responses triggered by photostimulation of LC fibers in cortical slices.
- **Supplementary Figure 5**. Test of different patterns of LC fibers photostimulation *in vivo.*
- **Supplementary Figure 6.** LC fibers photostimulation *in vivo* does not trigger TurboRFP fluorescence changes.
- **Supplementary Figure 7.** Responses of individual dendritic ROIs to photostimulation of LC fibers *in vivo.*
- **Supplementary Figure 8.** Responses of individual somatic ROIs to photostimulation of LC fibers *in vivo.*
- **Transparent methods (pages 8-12)**

---

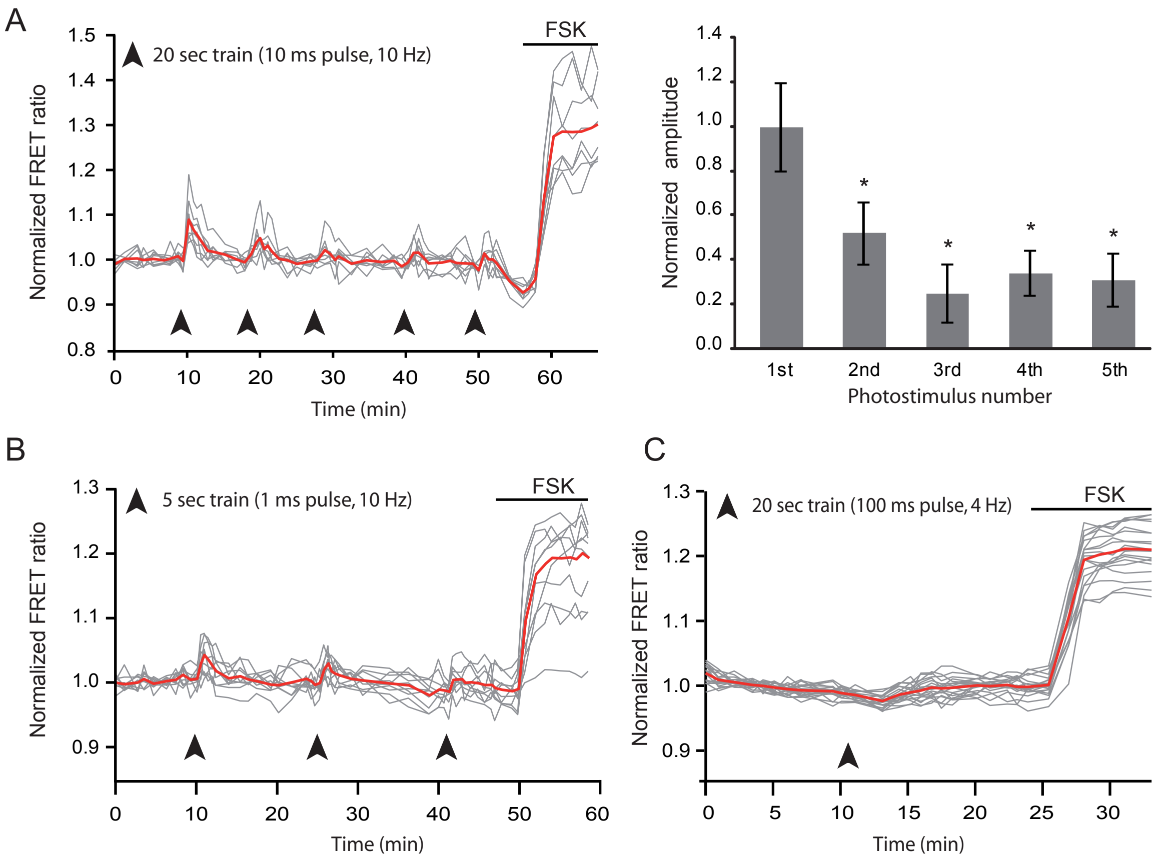


**Supplementary Figure 1. Test of different patterns of LC fibers photostimulation in cortical slices (related to figure 2).**

Two-photon imaging of layer V pyramidal neurons expressing the AKAR3EV sensor. Traces show the F535/F480 emission ratio measured at the soma of individual cells (grey traces, mean trace in red) upon burst photostimulation (arrowheads) of indicated patterns, and upon bath application of forskolin (FSK, 12.5 µM). (**A**) Data obtained in n=8 pyramidal neurons from N=1 slice. Note the decrease of response amplitude upon repetitive stimulation. * significantly different from the response to the first photostimulation. (**B**) Data obtained in n=9 cells, N=1 slice. Photostimulation elicited a 3.4 ± 0.4 % increase in ΔR/R0. (**C**) Data obtained in n=19 cells, N=2 slices. Note the absence of response, presumably related to ChR2 desensitization during 100 ms light pulses.

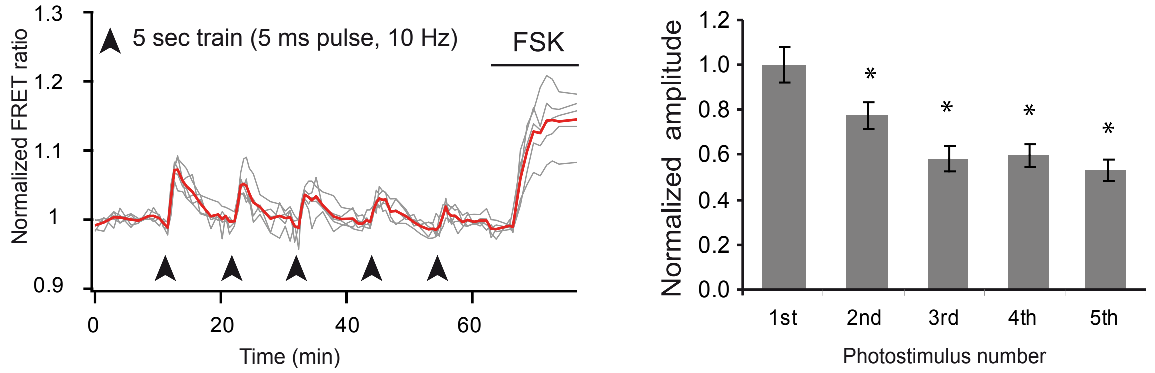


**Supplementary Figure 2. Responses of layer II/III pyramidal neurons to photostimulation of LC fibers in cortical slices (related to figure 2 and table 1).**

Two-photon imaging of layer II/III pyramidal neurons expressing the AKAR3EV sensor. Traces show variations of the F535/F480 emission ratio measured at the soma of individual pyramidal neurons (n=5, grey traces, mean trace in red), in response to burst photostimulation (arrowheads) and to bath application of forskolin (FSK, 12.5 µM). The bar graph summarizes results obtained in n=34 cells from N=4 slices, with differences from the response to the first photostimulation being statistically significant.

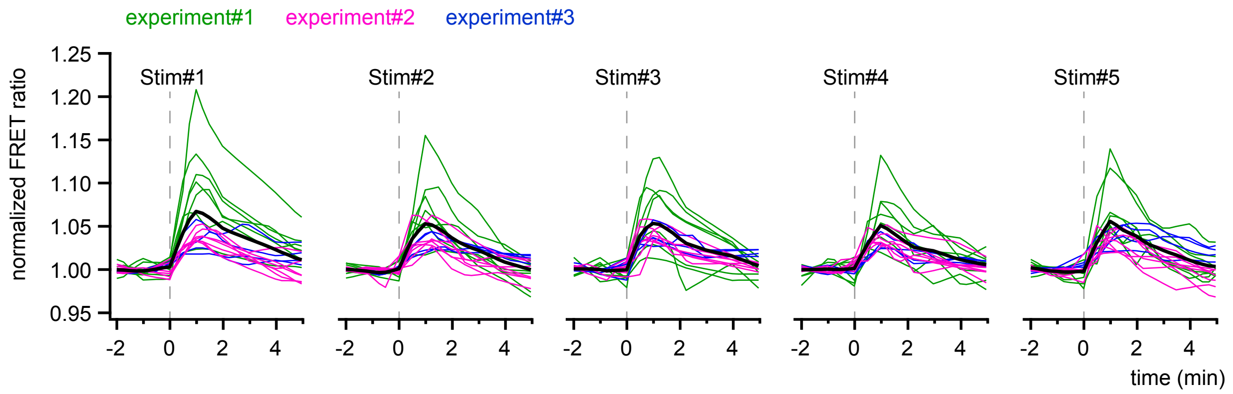


**Supplementary Figure 3. Variability of layer V pyramidal neurons responses to photostimulation of LC fibers in cortical slices (related to figure 2).**

Two-photon imaging of layer V pyramidal neurons expressing the AKAR3EV sensor. Traces show the F535/F480 emission ratio measured at the soma of individual cells (n=18, from 3 experiments as indicated, mean trace in black). Green traces correspond to the experiment shown in Fig. 2A). To compensate for variable baseline drifts during the course of the experiments, individual traces were realigned by normalizing to the baseline value measured before each stimulation trial.

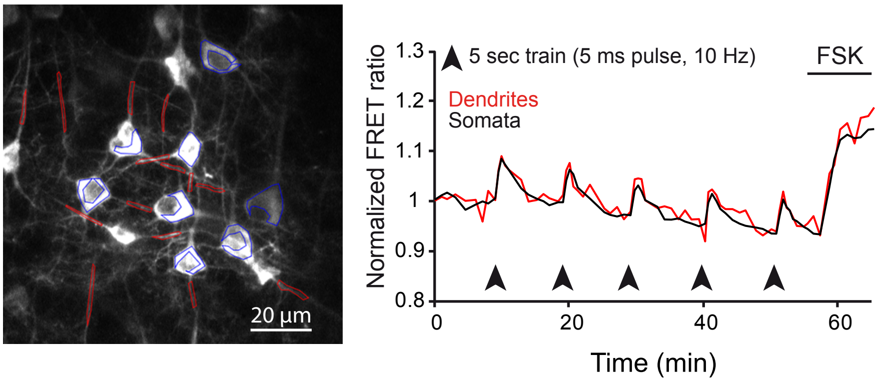


**Supplementary Figure 4. Comparison between dendritic and somatic responses triggered by photostimulation of LC fibers in cortical slices (related to figure 2).**

**Left:** Two-photon imaging of neuronal somata and dendrites expressing the AKAR3EV sensor in layer V. The greyscale image shows the F535 intensity and dendritic (red) or somatic (blue) ROIs delineated for measurements of F535/F480 emission ratio. **Right:** Traces show mean variations of the F535/F480 emission ratio measured at these somatic (n=8, black) and dendritic (n=14, red) ROIs in response to burst photostimulation (arrowheads) and to bath application of forskolin (FSK, 12.5 µM).

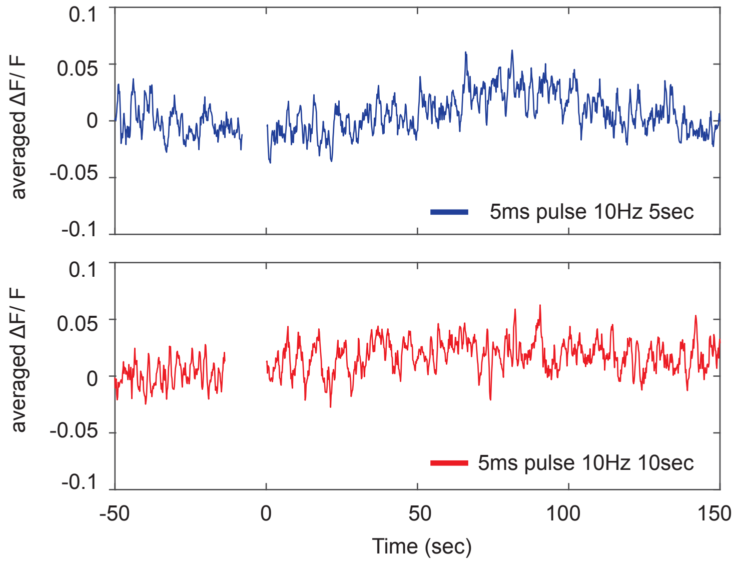


**Supplementary Figure 5. Test of different patterns of LC fibers photostimulation *in vivo* (related to figure 3).**

Two-photon imaging of cortical neurons expressing the GAkdYmut sensor. Traces show averaged GAkdYmut fluorescence intensity of 50 dendritic ROIs before and after photostimulation of indicated patterns delivered during the recording shut off intervals. Note the difference with clear responses shown in Fig. 3.

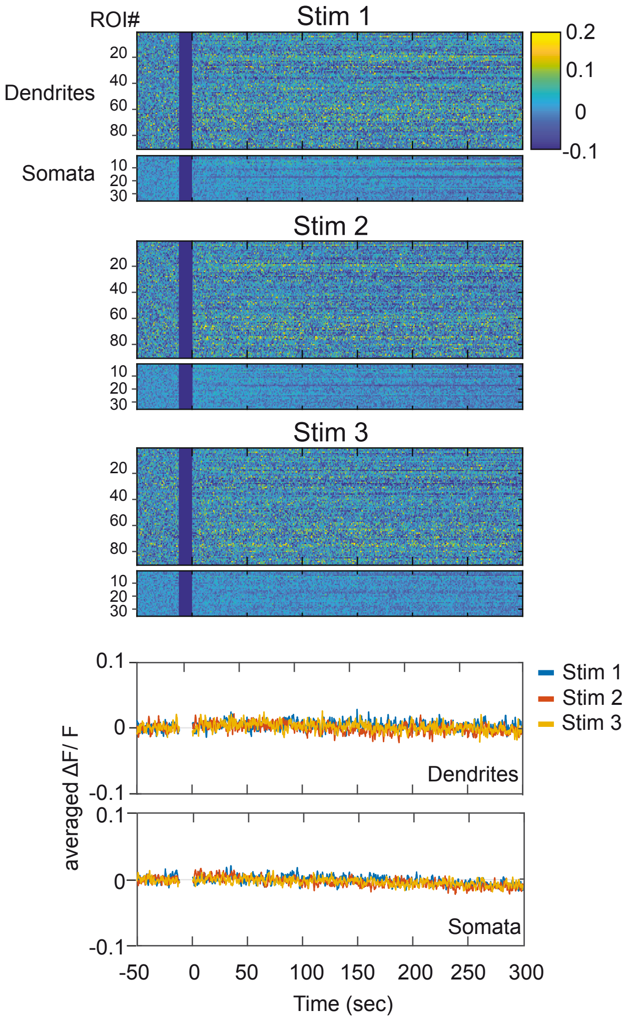


**Supplementary Figure 6. LC fibers photostimulation *in vivo* does not trigger TurboRFP fluorescence changes (related to figure 3).**

Two-photon imaging of cortical neurons co-expressing TurboRFP and the GAkdYmut sensor. ***Upper***: Raster plots show TurboRFP fluorescence intensity (calibration bar: ΔF/F) of dendritic and somatic ROIs corresponding to GAkdYmut recordings shown in Fig. 3A, before and after each of three consecutive photostimulation trials (darker vertical bar, 5 ms pulses at 20 Hz for 10 s). ***Lower***: Traces show averaged fluorescence intensity of the same ROIs.

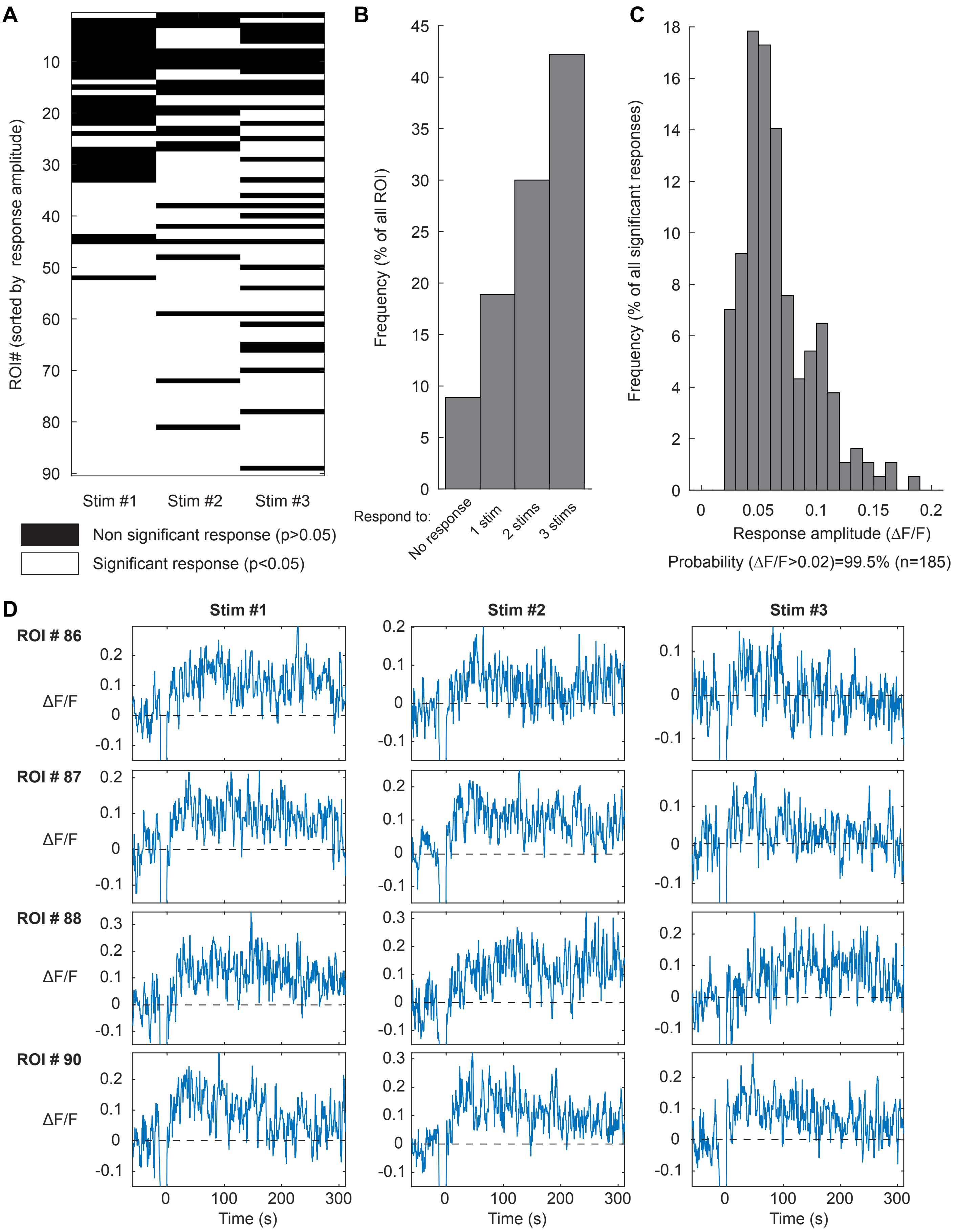


**Supplementary Figure 7. Responses of individual dendritic ROIs to photostimulation of LC fibers *in vivo* (related to figure 3).** (**A**) Rasterplot showing responsiveness of 90 dendritic ROIs arranged from top to bottom by increasing response amplitude upon three successive photostimulation trials (stim). ROI responsiveness was determined by comparing the fluorescence signals before and after stimulation using a Kolmogorov-Smirnov statistical test (see Methods). (**B**) Distribution of the same individual ROIs as a function of their number of responses to the three stimulation trials. (**C**) Distribution of amplitudes of all statistically significant responses of the same ROIs across the three stimulation trials. 99.5% of these significant responses corresponded to fluorescence increases larger than 2 % of baseline. (**D**) Fluorescence traces of the four ROIs exhibiting the largest responses to the three stimulation trials (t=0, end of the stimulus). The signal was smoothed by median filtering using a 20 datapoint sliding window.

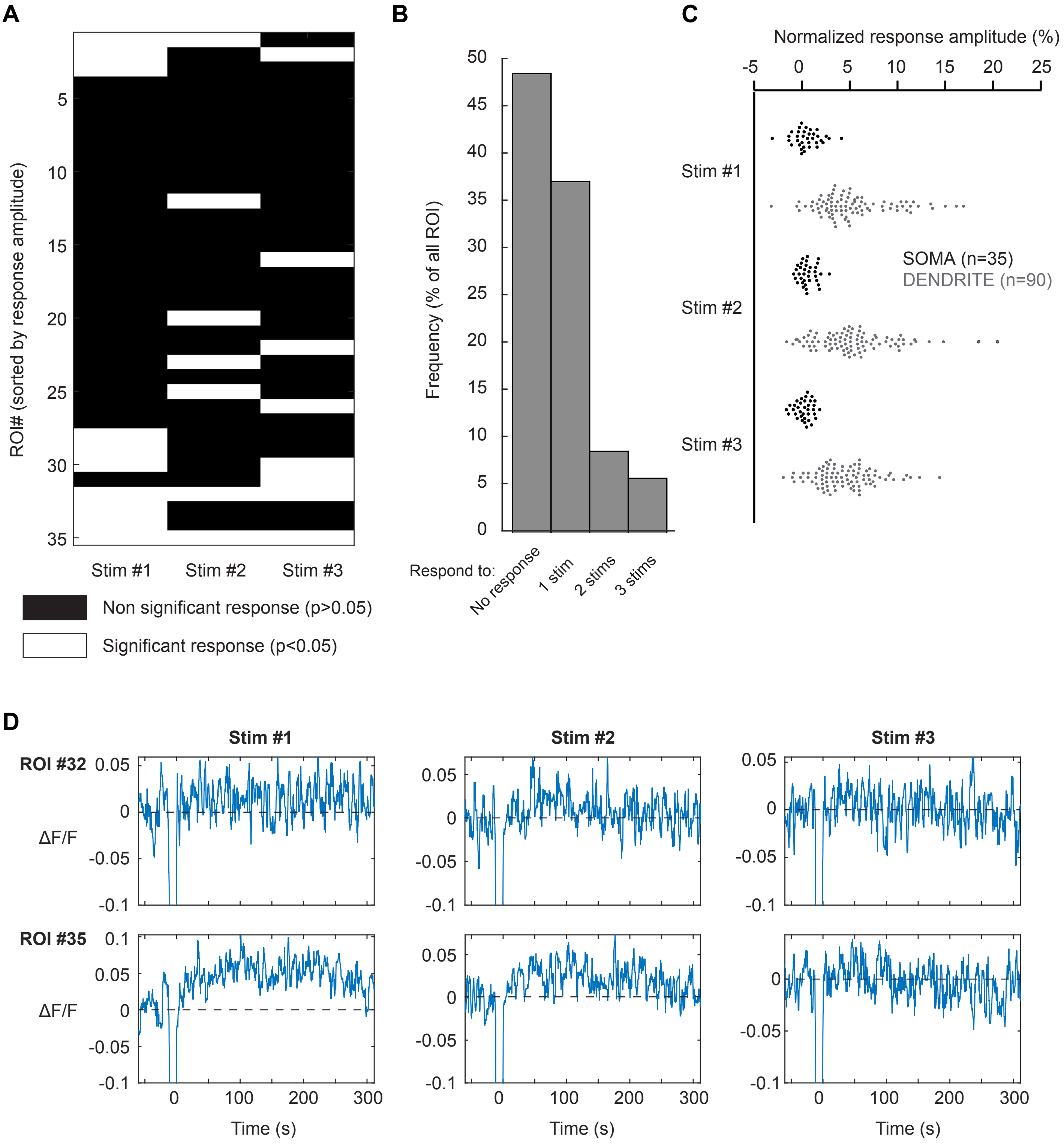


**Supplementary Figure 8. Responses of individual somatic ROIs to photostimulation of LC fibers *in vivo* (related to figure 3).** (**A**) Rasterplot showing responsiveness of 35 somatic ROIs arranged from top to bottom by increasing response amplitude upon three successive photostimulation trials (stim). ROI responsiveness was determined by comparing the fluorescence signals before and after stimulation using a Kolmogorov-Smirnov statistical test (see Methods). (**B**) Distribution of the same individual ROIs as a function of their number of responses to the three stimulation trials. (**C**) Fluorescence changes normalized to baseline in individual somatic and dendritic ROIs upon three successive photostimulation trials. Dendritic fluorescence changes were larger than somatic ones. (**D**) Fluorescence traces of the two somatic ROIs showing statistically significant responses to the three stimulation trials (t=0, end of the stimulus). The signal was smoothed by median filtering using a 20 datapoint sliding window.

## Transparent Methods

### Animals

Experiments were in accordance with the European Communities Council Directive 86/609/062, approved by the Charles Darwin ethics committee (project 01907.1) and the OIST Institutional Animal Care and Use Committee, and performed in accredited facilities. Transgenic DBH-Cre mice were a gift from Bruno Giros [MMRRC line: Tg(Dbh-cre)KH212Gsat/Mmucd, stock number 032081-UCD (Gong et al. 2007)]. Genotyping of DBH-Cre mice was done by PCR with primers: forward AATGGCAGAGTGGGGTTGGG, reverse CGGCAAACGGACAGAAGCATT (225 bp). Animals were maintained in a 12 h light–12 h dark cycle, in stable conditions of temperature (22°C), with food and water available *ad libitum*. Data were collected from 29 mice aged 5-7-weeks at the time of first viral injection, including 9 females and 13 males for brain slice experiments, and 7 females for *in vivo* experiments.

### Recombinant viruses

The adeno-associated virus (AAV) driving Cre-dependent expression of a fusion protein containing channelrhodopsin 2 (ChR2) and the yellow fluorophore YFP (AAV2/1-EF1a-DIO-hChR2(H134R)-EYFP-WPRE-HGHpA) was produced from Addgene plasmid #20298 (a gift from Karl Deisseroth) at vector core facilities of Nantes University (France, titer: $3x10^{11}$ gc/ml) and Pennsylvania University (USA, titer: $8x10^{12}$ gc/ml). The Sindbis virus encoding the AKAR3EV PKA activity sensor (Komatsu et al. 2011) was produced as described (Hepp et al. 2007, SIN-AKAR3EV titer: $1x10^{9}$ ip/ml) and mixed with a Sindbis virus encoding the red fluorophore mCherry (SIN-mCherry, gift from Katia Boutourlinski, UMR8246 Paris) at a ratio 40:1. The AAV encoding the GAkdYmut PKA activity sensor (Bonnot et al. 2014) was custom made and produced by the vector core facility of Pennsylvania University (AAV2/1-hSyn-GAkdYmut-hGH, titer: $4x10^{14}$ gc/ml), and mixed with AAV2/1-hSyn-TurboRFP-WPRE encoding the red fluorophore TurboRFP (titer: $4x10^{13}$ gc/ml, same supplier) at a ratio 1:1.

### Expression of ChR2 and AKAR3EV for optogenetic experiments in cortical slices

Site- and cell-type-specific expression of ChR2 in NA neurons was achieved by viral transfer into the LC of DBH-Cre mice, exactly as described (Nomura et al. 2014). Mice (1-month-old) were anesthetized with isoflurane and the AAV-ChR2-YFP suspension (titer: $3x10^{11}$ gc/ml) injected bilaterally at coordinates from bregma: anteroposterior (AP) -5.45 mm, mediolateral (ML) ± 1 mm, dorsoventral (DV) -3.65 mm, through an internal canula at a rate of 0.1 µl/min for 10 min (1 µl/site). For postoperative care, ropivacaine (10 mg/ml) was added dropwise on the skin suture, and mice received buprenorphine (0.1 mg/kg). Eight to ten weeks after AAV-ChR2-YFP injection, mice were

anesthetized with 2 % isoflurane and the SIN-AKAR3EV/SIN-mCherry viral suspension mix was injected bilaterally in the primary sensory area of the parietal cortex at coordinates from bregma: AP -1.5 mm, ML ± 2 mm, DV -0.4-0.5 mm, through a glass pipette at a rate of 0.1 µl/min (2 µl/site). Mice received postoperative care as described above. Ten to twelve hours after Sindbis virus injection, mice were deeply anesthetized with 10 mg/kg ketamine and 0.1 mg/kg xylazine before transcardiac perfusion with 20 ml ice cold high-sucrose/low-calcium artificial cerebrospinal fluid (ACSF) containing: 73 sucrose, 85 NaCl, 2.5 KCl, 1.25 NaHPO4, 0.4 $CaCl_2$, 7 $MgCl_2$, 26 $NaHCO_3$, 20 D-glucose, 5 Na pyruvate, 2 kynurenic acid (mM), 50 nM minocycline hydrochloride (Tikka et al. 2001), saturated with 5% $CO_2$/95% $O_2$. The brain was dissected and coronal slices (300 µm thick) of neocortex were cut in high-sucrose/low-calcium ACSF using a VT1000S Vibratome (Leica). Slices were kept at 33°C for 30 min in ACSF containing 126 NaCl, 2.5 KCl, 1.25 $NaHPO_4$, 2 $CaCl_2$, 1 $MgCl_2$, 26 $NaHCO_3$, 20 D-glucose, 5 Na pyruvate (mM), supplemented with 2 mM kynurenic acid and 50 nM minocycline hydrochloride, and bubbled with 5% $CO_2$/95% $O_2$. Slices were then allowed to recover at room temperature for 1 hour. Slices were transferred into a recording chamber perfused with ACSF (2 ml/min, 30 °C, pH 7.4) bubbled with 95% O2 and 5% CO2. Slices were inspected for mCherry expression using wide-field fluorescence to localize viral transduction area with minimal excitation of ChR2.

### Expression of ChR2 and GAkdYmut for optogenetic experiments *in vivo*

ChR2 was expressed in NA neurons essentially as described above. The AAV-ChR2-YFP suspension (titer: $8x10^{12}$ gc/ml) was injected bilaterally into the LC of 5 to 7-week-old DBH-Cre mice at a rate of 10 nl/min (350 nl/site). Three weeks after AAV-ChR2-YFP injection, anesthesia was induced with a mixture of medetomidine (0.3 mg/kg), midazolam (4 mg/kg) and butorphanol (5 mg/kg), and a 5 mm craniotomy was performed under 2% isoflurane anesthesia. Then, 70-140 nl of the AAV-GAkdYmut/AAV-TurboRFP suspension mix was injected in layer V of the parietal cortex at coordinates from bregma: AP -1.5 mm, ML 1.7 mm, DV -0.6-0.7 mm, at a rate of 10 nl/min. A chronic cranial window (5 mm glass coverslip) was next mounted as described (Roome and Kuhn 2014). At the end of the surgery, mice received atipamezole (0.3 mg/kg) for recovery from anesthesia, and postoperative care as described above. Five to eight weeks after the last AAV injection, mice were head-fixed for imaging experiments performed under anesthesia with 1% isoflurane.

### Optical stimulation and imaging in cortical slices

Two-photon images were obtained with a custom built two-photon laser scanning microscope described earlier (Castro et al. 2010, Bonnot et al. 2014), and using a 60X (0.9 NA, Olympus) water-immersion objective. Sampling rate was at 1.4 frame / s. For imaging of AKAR3EV, based

on fluorescence resonance energy transfer (FRET) between a blue donor (ECFP) and a yellow acceptor (YPet), 2-photon excitation of ECFP was performed at 850 nm with a power of 30 mW. ECFP and YPet fluorescence signals (peaks 480 and 535 nm, respectively) were filtered (Chroma Technology: E700 SP, Semrock: FF01-479/40, FF01-542/50), separated with a dichroic beamsplitter (FF506-Di02-25x36 Semrock) and simultaneously detected in two channels (H9305 photomultipliers, Hamamatsu). Images were acquired every minute prior to ChR2 photostimulation, and then every 30 s following photostimulation.

ChR2 photostimulation consisted in 470 nm light pulses delivered by a LED (LEDD1B, Thorlabs) through a 400 µm diameter fiber optic cable (ThorLabs) controlled with micromanipulators (ROE-200, Sutter Instrument). The light power at the tip of the optical fiber was 10 mW. The fiber tip was placed just above the slice surface and positioned 400 µm away from the center of the imaging field with an angle of 30° from the slice surface. Light pulses and a shutter (DSS25 and VDM1000, UNIBLITZ) protecting photomultipliers from light interference during photostimulation were controlled by custom software written in LabVIEW (National Instruments).

The 512x512-pixel images were exported using MATLAB (The Mathworks), and analyzed with custom macros derived from ImageJ (U.S. National Institute of Health). Fluorescence intensity of regions of interest (ROIs) was calculated for each time point from average intensity z-projection of 3 frames by averaging pixel intensity. Fluorescence changes were measured as the ratio R=F535/F480. Pseudocolor hue saturation value (HSV) encoding of fluorescence intensity and ratio was performed using MATLAB custom procedures written by Hirokazu Tanimoto (Yokohama City University). Decay time-constants were obtained using the exp-XOffset fit function of IgorPro6 (WaveMetrics) using the equation: $y_0 + Aexp\left\{\frac{-(x-x_0)}{\tau}\right\}$

**Optical stimulation and imaging in the cortex *in vivo***

A combined wide-field/two-photon microscope (MOM, Sutter Instruments) with a femtosecond-pulsed Ti:sapphire laser (Vision II, Coherent) was used. To increase the point spread function of excitation the back aperture of the 25X water immersion objective (Olympus) was underfilled (spatial resolution 1 µm x 1 µm x 4 µm). The collar of the objective was adjusted to correct for the window glass thickness (170 µm). Simultaneous excitation of GAkdYmut (GFP-based single fluorophore sensor, Bonnot et al. 2014) and TurboRFP was performed at 950 nm with a typical power of 5-11 mW. Fluorescence was detected in two channels by GaAsP photomultipliers (Hamamatsu) in spectral windows 490-550 nm (GAkdYmut) and 600-700 nm (TurboRFP), separated by a 560 nm dichroic mirror (all Semrock). The microscope was controlled by a commercial software (MScan, Sutter Instruments). Sampling rate was 30.9 frame/s.

ChR2 photostimulation was done with a collimated blue LED (450-495 nm, BDX, X-Cite XLED1,

Lumen Dynamics) illuminating the area of the chronic cranial window with an angle of 30° from the glass surface. The power at the window glass surface was 0.35 mW/mm². Photomultipliers were manually shut off during photostimulation.

Movies (512 x 512 pixel per frame, corresponding to a field of view of 375 µm x 375 µm) were analyzed with custom made MATLAB code and ImageJ. The 30.9 Hz imaging data was temporally binned to 6.2 Hz. ROIs were selected from the GAkdYmut channel and relative fluorescence change $\frac{\Delta F}{F}(t)$ was calculated as: $\frac{\Delta F}{F}(t) = \frac{F(t)-F_{baseline}}{F_{baseline}}$ , where $F_{baseline}$ is the average fluorescence intensity during a 30 s interval ending 5 s before photostimulation. Onset and decay time-constants were obtained by fitting responses with the function: $A\left[exp\left(-\frac{t}{\tau decay}\right) - \exp\left(-\frac{t}{\tau\ onset}\right)\right] + B$

**Histology**

The expression of ChR2-YFP and dopamine-β-hydroxylase was examined on fixed brain slices using a polyclonal rabbit anti DBH (1/1000, AB1538, Merck-Millipore) and a polyclonal chicken anti-GFP antibody (1/2000, GFP-1020, Aves Labs), as described (Nomura et al. 2014). Secondary antibodies were Alexa Fluor 488 goat anti chicken (1/1000, Life Technologies A11039) and goat anti Rabbit-Alexa 555 (1/1000, Life Technologies A-21428). Slices were mounted in fluoromount-G (Clinisciences). Images were acquired using a confocal laser scanning microscope (SP5, Leica) or using wide-field epifluorescence (DMR microscope, Leica) and processed with ImageJ. Following *in vivo* imaging experiments, brains were fixed, sliced in 60 µM sections, and images were acquired using a wide-field fluorescence microscope (Nikon Eclipse, Nikon).

**Drugs**

Drugs were purchased from Tocris or Sigma-Aldrich. Drugs were bath applied on brain slices: CGP20712 hydrochloride (100 nM), SCH23390 hydrochloride (1µM); Yohimbine (1 µM,) Haloperidol (10 µM), Forskolin (12.5 µM), Reboxetine mysylate (100 nM), GBR12783 dihydrochloride (100 nM), Corticosterone (100 µM). For in vivo pharmacology, saline, Propranolol (4 mg/kg), Reboxetine mesylate (10 mg/ kg) were injected intraperitoneally.

**Statistical analyses**

In this report, N represents the number of brain slices or mice tested while n represents the number of cells or ROIs on which measurements were performed. Data are presented as mean ± standard error of the mean (SEM). Statistical analyses were performed with MATLAB. Unless otherwise stated, one-way ANOVA followed by Tukey‘s test was used to determine statistical

significance, except for analyses of single ROI responsiveness *in vivo*. To evaluate the responsiveness of individual ROIs *in vivo*, the fluorescence signal was binned 5 times to reach a 6.2 Hz sampling rate, and then normalized to the baseline calculated as the mean fluorescence intensity over the time window [-68.9;-20.4 s] (t=0 refers to the end of the photostimulation). Response amplitude measured over the interval [20.0;52.4 s] (n=201 data points) was then compared to baseline signal (301 data points) using a Kolmogorov-Smirnov test. Throughout this manuscript, a p-value below 0.05 was considered statistically significant.

**Supplemental references**


Gong, S., Doughty, M., Harbaugh, C.R., Cummins, A., Hatten, M.E., Heintz, N., and Gerfen, C.R. (2007). Targeting Cre recombinase to specific neuron populations with bacterial artificial chromosome constructs. J. Neurosci. 27, 9817-9823.

Hepp, R., Tricoire, L., Hu, E., Gervasi N., Paupardin-Tritsch, D., Lambolez, B., and Vincent, P. (2007). Phosphodiesterase type 2 and the homeostasis of cyclic GMP in living thalamic neurons. J. Neurochem. 102, 1875-1886.

Roome, C.J., and Kuhn, B. (2014). Chronic cranial window with access port for repeated cellular manipulations, drug application, and electrophysiology. Front. Cell. Neurosci. 8, 379.

Tikka, T., Fiebich, B.L., Goldsteins, G., Keinanen, R., and Koistinaho, J. (2001). Minocycline, a tetracycline derivative, is neuroprotective against excitotoxicity by inhibiting activation and proliferation of microglia. J. Neurosci. 21, 2580-2588.
